# How is practitioner-led quality and safety improvement sustained across a whole hospital? A process evaluation of a practice development programme

**DOI:** 10.3389/frhs.2026.1932480

**Published:** 2026-10-14

**Authors:** Jacqueline Peet, Karen Tuqiri, Ruth Smoother, Suzanne Sheppard-Law, Clint Douglas

**Affiliations:** 1 School of Health, University of the Sunshine Coast, Brisbane, QLD, Australia; 2 Caboolture, Kilcoy and Woodford Directorate, Metro North Health, Brisbane, QLD, Australia; 3 The Prince of Wales Hospital, South Eastern Sydney Local Health District, Sydney, NSW, Australia; 4 School of Nursing and Midwifery, University of Technology Sydney, Sydney, NSW, Australia; 5 School of Nursing, Queensland University of Technology, Brisbane, QLD, Australia; 6 Office of Nursing and Midwifery Services, Metro North Health, Brisbane, QLD, Australia

**Keywords:** facilitation, hospital care, nursing, practice development, process evaluation, quality improvement, safety culture

## Abstract

**Background:**

Building frontline workforce capability to sustain quality and safety improvement across whole hospitals is a strategic priority for healthcare leadership. The principles are well established; how to enact and sustain them at scale is not. Practice development, a participatory approach to workforce transformation, has not been evaluated at hospital-wide scale.

**Objectives:**

To develop and refine programme theories explaining how and why a hospital-wide practice development programme for quality and safety produces its effects in different settings, and to identify the conditions under which practice development mechanisms work, and fail to work, at hospital-wide scale.

**Methods:**

This study was a qualitative process evaluation at a major Australian tertiary referral hospital where nursing leaders implemented a Quality Safety Culture Framework across all 41 acute-care units over 6 years. Practice development theory provided the sensitising framework; the empirical material was used to challenge rather than confirm its assumptions. Fieldwork comprised 27 interviews with nursing, medical, allied health, and executive staff; 6 half-day workshops with 49 frontline staff; a multidisciplinary focus group; and approximately 20 h of observations. Four tentative programme theories were developed prior to analysis. Abductive analysis foregrounded theoretical surprise through sustained dialogue between fieldwork and existing theory; within-programme variation provided the analytical structure.

**Findings:**

The same programme simultaneously produced sustained participatory quality and safety improvement in some settings and directive compliance in others. Five refined programme theories specify the conditions for enactment: how data are used for learning rather than accountability; whether communicative space is sustained as an organisational precondition; what facilitators actually do; whether leadership creates organisational enablement without prescribing what fills it; and how collaborative prioritisation prevents project proliferation from undermining engagement. Implementation drift from participatory towards directive processes was concentrated in the facilitation pipeline, driven by governance logics that systematically favour measurable quality improvement outputs over relational and cultural processes.

**Conclusions:**

Organisational enablement is necessary but insufficient to sustain participatory practice. Even with genuine participatory design intent, governance infrastructure built to reward measurable outputs can systematically displace participatory processes. The five theories specify the conditions required to sustain participatory approaches at scale and offer practical guidance for hospital leadership teams.

## Introduction

Frontline nursing teams are central to delivering safe, person-centred care in acute hospitals, yet how to transform nursing workforce capability for quality and safety at an organisational scale remains unclear (1). Hospital-acquired complications remain a leading cause of preventable harm, and staff experience of workplace culture directly shapes patient outcomes (2). Quality improvement (QI) interventions are typically developed and tested locally, in individual units or around specific safety problems, and this is where improvement work should begin. Building the workforce capability to generate, lead, and sustain quality and safety improvement from the point of care—across whole hospitals—is a different undertaking: a strategic priority for healthcare leadership, but one that lacks an evidence base for how it is achieved in practice (3, 4).

Over the past 6 years, nursing leaders at a major Australian tertiary referral hospital have implemented an organisation-wide Practice Development (PD) programme for quality and safety across 41 acute-care units. Known locally as the Quality Safety Culture Framework (QSCF), the programme targets frontline teams where care is experienced and provided, with organisational leaders playing a crucial enabling role. It focuses on developing clinical leadership for safety and facilitation skills for person-centred practice change. As the largest programme of its kind in Australia, it presents a unique opportunity to examine how practitioner-led transformation operates at hospital-wide scale.

## Background

PD has undergone substantial theoretical maturation over the past decade, evolving from microsystem-level accounts of culture change towards increasingly ambitious frameworks for whole-system transformation (5). Hardy et al. (6) refined PD's core methodological principles, presented in Figure 1. Grounded in critical social science, PD uses facilitation, active work-based learning, and critical inquiry to foster communicative action oriented towards mutual understanding rather than strategically influencing others to achieve predetermined ends—transforming workplace cultures from within through person-centred, collaborative and participatory approaches (7).

**Figure 1:**
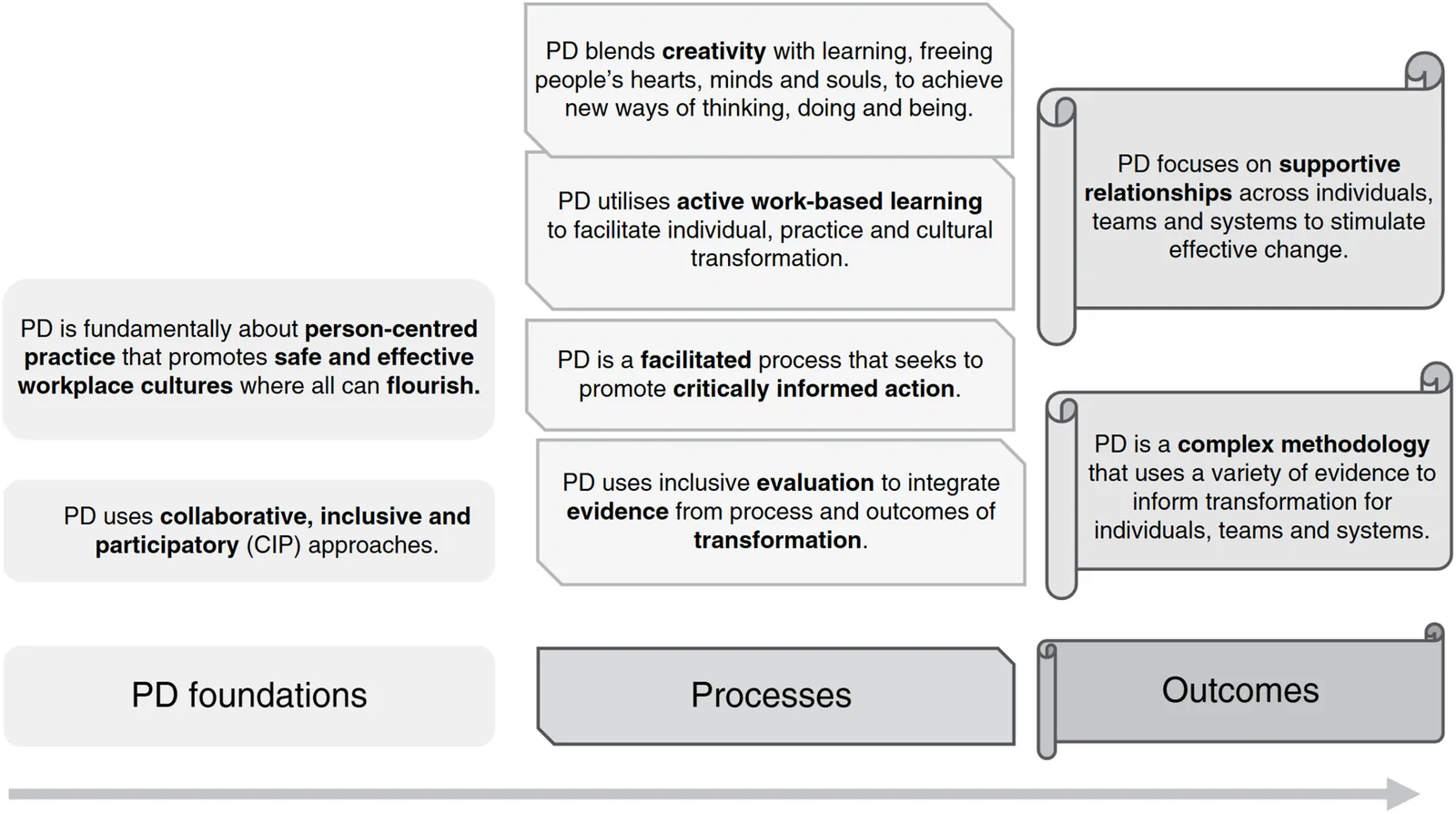
Practice development principles (6). © 2021 John Wiley & Sons Ltd. Reprinted with permission.

Building on these foundations, Manley et al. (8) refined a PD theory of culture change at the microsystem level through the SCQIRE (Safety Culture Quality Improvement Realist Evaluation) project, a realist evaluation across 10 frontline teams in four NHS trusts. A key finding was that safety cultures cannot develop without person-centred relationships being experienced by both staff and patients—the value clusters of person-centredness, ways of working, and effective care are interdependent.

PD research in acute-care settings has shown that emancipatory PD can build safety cultures at the microsystem level through communicative action and practitioner-led innovation (9, 10). PD theory has since expanded beyond the microsystem. The Venus model (3) synthesised four large-scale PD studies into five interdependent stems—skilled facilitation, leadership, practice development, improvement, and culture change—positioning transformation as requiring all five stems to work together at micro-, meso-, and macro-levels. Most recently, the SHIFT (Sustainable Health and Care Innovation for Future Transformation) framework (11) connected micro-level culture change to system-level outcomes through a meta-analysis of 46 PD projects over 12 years.

However, these frameworks were developed through the synthesis of individual PD projects and collaborative initiatives. They describe what PD should achieve across healthcare systems, but the conditions under which PD's mechanisms operate when enacted across a whole hospital—simultaneously, over years, under the governance pressures of accreditation standards, performance accountability, and managerialist healthcare—have not been studied in practice. The broader conditions for large-scale system transformation have been identified as system enablers (12, 13), but these frameworks do not specify what fills the space that enablers create.

### The relationship between PD and quality and safety improvement

How PD relates to quality improvement in acute-care settings remains unresolved. Lavery (14) proposed PD and QI as allies, assuming symmetry between the approaches—shared goals, complementary methods. Manley et al. (15) cautioned against this framing, warning of synecdoche: the risk that QI's measurable process improvements are mistaken for the broader organisational transformation PD pursues. In PD, structures and processes follow values and purposes, not vice versa (16). More recently, Reed et al. (17) outlined the landscape of improvement science in terms that absorb much of PD's traditional territory—person-centredness, relational dimensions of change, complex systems thinking—without naming PD as a distinct tradition. The field has moved from Lavery's allies-or-rivals question towards a more fundamental issue PD theory has not addressed: what determines whether participatory or directive processes fill the organisational space created for quality and safety improvement?

By participatory processes we mean PD's collaborative, inclusive, and participatory principles enacted in practice (6): practitioner-led transformation in which frontline teams identify their own practice priorities, generate and test solutions, and make sense of evidence through facilitated collective action. By directive processes we mean those in which priorities, methods, and reporting requirements are determined elsewhere and the unit's role is compliance. The distinction is observable: who sets the agenda, who does the analytical work, and whether facilitated collective action opens deliberation or delivers instruction.

This question is sharpened by research on facilitation. Kislov et al. (18) demonstrated three processes through which facilitation is distorted over time in healthcare settings: prioritisation of measurable outcomes over interactive process, reduction of team engagement, and erosion of the facilitator role. Critically, facilitator agency was constrained by the very governance influences facilitators attempt to mitigate. Peet et al. (9) showed what emancipatory PD achieves at the microsystem level—frontline nurses building safety cultures through values clarification, communicative action, and practitioner-led innovation—but reflected on the challenges of facilitating change without structural change in the outer context, and how PD might contribute alongside other organisational change approaches (9, 10). Greenhalgh et al.'s (19) participatory continuum for organisational innovation—“let it happen,” “help it happen,” “make it happen”—provides a useful shorthand for locating programmes on this spectrum and detecting drift. The programme under evaluation was explicitly designed as “help it happen.” Whether and how this positioning was maintained across six years and 41 clinical units is a question this study addresses.

### Study aim

This process evaluation aimed to

- develop and refine programme theories (PTs) explaining how and why the QSCF produces its effects in different settings and
- identify the conditions under which PD's mechanisms work, and fail to work, at a hospital-wide scale.

## Materials and methods

### Study design

This qualitative process evaluation is aligned with the Medical Research Council framework for developing and evaluating complex interventions (20). The evaluation is informed by realist evaluation's commitment to refining programme theories through examining contextual variation (21). Our analytical approach was abductive (22, 23), foregrounding theoretical surprise (23) and the construction of an analytical argument rather than the systematic testing of predetermined context–mechanism–outcome configurations. The programme theories are realist in form and abductive in derivation. Realist evaluation requires a generative account that begins and ends with programme theory; abduction provides the analytical tools for theoretical refinement.

The approaches outlined above share a commitment to theoretically informative research (5, 24) where the analytical endpoint is a theoretical argument constructed through sustained dialogue between empirical material and existing scholarly understanding—not a set of inductive themes. PD theory (3, 6, 8) provided the sensitising framework: the theoretical vocabulary through which patterns were identified, interrogated, and rendered surprising. The broader literatures on facilitation and participatory implementation (18, 19) constituted additional theoretical resources through which the empirical material was engaged.

The refined programme theories are explanatory propositions in the realist tradition (21): claims about generative mechanisms and the conditions under which they tend to operate, justified by the coherence of the theoretical argument rather than by successionist causal inference. Consistent with this tradition, we assumed that programme success is always partial; the analytical task was to explain how and why the programme produces its effects across contexts, not to adjudicate whether it works.

### Programme context

The Quality Safety Culture Framework was established at this major metropolitan hospital by the Director of Nursing (DON) in 2019 and implemented across all 41 acute-care units over 6 years. The hospital sits within a local health district with a 20-year history of PD and a district-wide strategy for person-centred practice (25), a deep contextual precondition for the QSCF.

The framework situated PD action cycles informed by quality improvement processes (Figure 2). Core components included: daily safety huddles across all settings; quality and safety boards in every work unit; a Clinical Leads facilitation programme supporting approximately 185 clinicians across eight high-risk clinical domains; audits of practice aligned to National Safety and Quality Health Service Standards; an annual hospital-wide Safety Attitudes Questionnaire (SAQ) with facilitated unit-level feedback (26); and the Real Time Patient Experience Survey. The work unit “culture map” integrates staff and patient experience data with clinical safety indicators and workplace observations to generate context-specific evaluations of safety culture.

**Figure 2:**
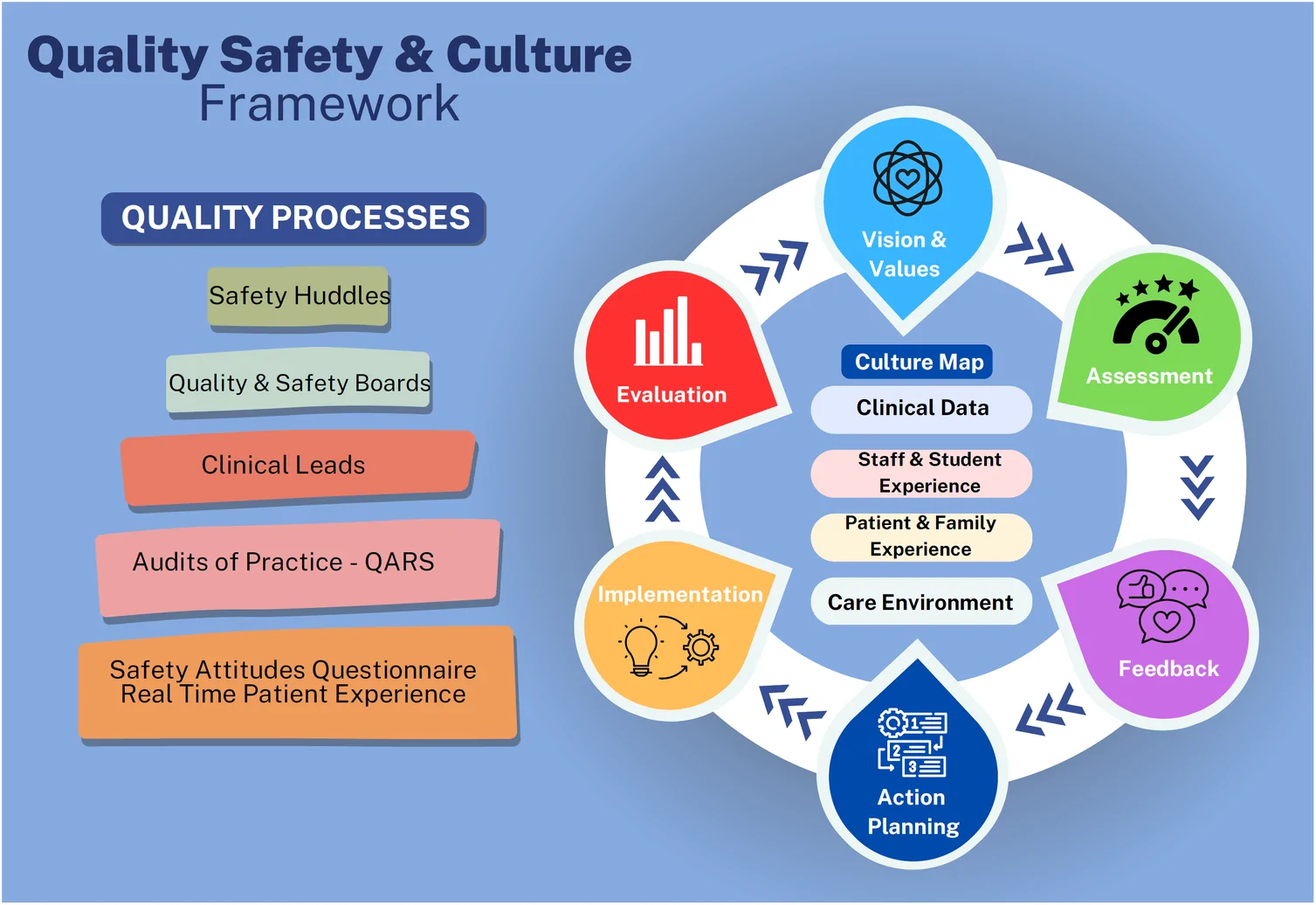
Quality Safety Culture Framework. Reproduced from https://doi.org/10.6084/m9.figshare.33684364 under a CC BY 4.0 licence.

The programme drew on the NSW Health Essentials of Care framework, a state-wide PD programme (27), the Clinical Excellence Commission's Building Blocks for a Safe Ward (28), and the Person-Centred Practice Framework (29). It was designed by nursing leaders as what Greenhalgh et al. (19) term “help it happen”: practitioner-led, participatory, with organisational enablement rather than a top-down mandate.

### Tentative programme theories

Four tentative PTs using simple if–then formulations were developed collaboratively with the research team prior to analysis, informed by PD theory. These represented the leadership team's initial espoused theories about how and why the QSCF worked to produce its effects.

***Evaluation (PT1):*** IF frontline teams evaluate their practice based on what matters to staff and patients (culture map) and have clear guidance to develop safety indicators and practices (huddles, boards, audits of practice, and surveys), THEN collaborative and participatory discussion around patient safety occurs, supporting bottom-up team learning and challenging practice assumptions and power hierarchies.
***Engagement (PT2):*** IF teams come together and are facilitated to reflect on safety at ward and hospital levels, THEN a communicative space opens where staff make safety values visible to themselves and others, their contributions are recognised, and organisational learning is shared.
***Facilitation (PT3):*** IF clinical leads develop facilitation skills to enable their teams to improve clinical safety priorities, THEN facilitators catalyse local practice change through systematic cycles of learning, action, and reflection, enabling collaborative change and person-centred cultures.
***Context and Leadership (PT4):*** IF teams experience person-centred leadership at all levels, with role clarity and ongoing support from nursing leadership, THEN despite changing contexts and team membership, there is continuity of purpose and democratic team ways of working for practitioner-led quality and safety.

These tentative assumptions guided initial analytical engagement with the empirical material. The findings present the refined programme theories—including a fifth PT addressing collaborative prioritisation—showing where and how the evidence challenged, extended, or reframed each theory.

### Data generation

Fieldwork was conducted over six months across the hospital, in the programme's sixth year. We conducted 27 face-to-face interviews with nursing unit managers, clinical nurse consultants, nurse educators, clinical leads, medical and allied health staff, nursing co-directors, and hospital executives, purposively sampled across clinical settings. The interview topic guide (Supplementary File 1) invited accounts of moments when the programme worked in unanticipated ways, the unspoken rules shaping how teams engage with it, and occasions when facilitation did not go as planned. Sampling sought variation across programme roles, professional groups, and clinical settings, the contrast on which the analysis depends. Adequacy was judged by theoretical sufficiency: whether the material could sustain, challenge, and refine the programme theories across that variation (30). Saturation was not the criterion. Our analytical endpoint was a theoretical argument, not categories or themes (22, 23), so there was nothing to saturate (30).

In parallel, we co-facilitated (JP, CD, and SS-L) six half-day workshops with 49 frontline staff and a multidisciplinary focus group across the hospital's five clinical departments. The workshops enabled deeper team-based critical reflection on how the QSCF worked and where the espoused programme theories were enacted in practice. We conducted approximately 20 h of field observations (JP and CD), encompassing safety huddles, clinical leads workshops, ward visits, and SAQ feedback sessions. All interviews were conducted by external researchers not involved in programme delivery. Interviews and workshops were audio-recorded with consent and transcribed verbatim. Workshop data were captured through facilitator notes, transcripts, and participant-generated materials. Observations were documented with field notes. We also engaged with programme documents including the framework diagrams, culture maps, safety board photographs, and internal reports.

### Analysis

In abductive analysis, empirical material serves as a critical dialogue partner for new theoretical interpretations, “not as a judge or a mirror” on reality (22, p. 62). We read all sources whole and developed written analytical interpretations (JP and CD) of how programme theories worked, or failed to work, across different contexts (31). The tentative programme theories and PD's sensitising framework generated expectations against which the empirical material generated questions—what Alvesson and Kärreman (22) term breakdowns, and what Timmermans and Tavory (23) describe as surprises against a backdrop of theoretical familiarity. Breakdowns were pursued only where they withstood scrutiny, resisting explanation within PD theory (22). Several breakdowns proved analytically generative, each revealing a gap between the programme's espoused theory and its enactment in practice.

These breakdowns became the study's central analytical question: why does the same programme produce participatory improvement in some settings and directive compliance in others—within the same hospital, under the same governance conditions? The programme theories were refined through systematic examination of within-programme variation, comparing settings where mechanisms fired with settings where they did not. Successive drafts of the written interpretations documented this movement from tentative to refined theory. Case examples illustrate programme theory enactment using participant language grounded in the empirical material. Evidence of drift, and of settings where mechanisms did not fire, is also presented in the narrative findings surrounding these examples.

Rigour was strengthened through the use of within-programme variation as an analytical strategy; sustained dialogue between the empirical material and multiple theoretical literatures; collaborative theory-refinement with programme stakeholders who could challenge the analytical claims; and attention to negative cases where theorised outcomes were not observed.

### Team reflexivity

Two of us (JP and CD) conducted interviews, co-facilitated workshops and led the analysis. These overlapping roles shaped the material in ways we worked with rather than sought to remove: facilitating the workshops gave us direct experience of where the espoused programme theories met resistance in the room, while also positioning us as people to whom participants might present the programme favourably. Reading the workshop material against interviews, observations, and programme documents was one way we kept this reflexive tension in view.

The DON's positioning as both programme architect and co-investigator was managed through practical governance arrangements: she was not present during any fieldwork, did not have access to transcripts, and all interpretations were developed by the external researchers before collaborative theory refinement.

### Ethics

This study received ethical approval from the South Eastern Sydney Local Health District Human Research Ethics Committee (2025/ETH00710). All participants provided written informed consent. Recordings and field notes were de-identified at the time of transcription. Where potentially re-identifiable, vignettes were shared and written permission was obtained.

## Findings

The same programme, designed with genuine participatory intent, simultaneously produced sustained participatory quality and safety improvement in some settings and directive compliance in others. Five refined programme theories explain this within-programme variation, each specifying conditions under which PD's mechanisms operate at hospital-wide scale.

The question

*In 2019, the Director of Nursing gathered the hospital's nursing leadership from each unit and asked a single question: how do you know the care being provided in your unit is safe and person-centred? No one answered. Rather than filling the silence, she waited—“I’m OK with silence.” In that moment, the absence of an answer revealed the absence of a space in which quality and safety could be openly explored. The DON's response was not to provide answers but to create the conditions for dialogue: “when I ask this question again in six to twelve months, you’re going to have an answer. But you need to trust me and we need to draw a line in the sand with what we have previously done.” Over the following six years, she enabled public forums where quality and safety could be courageously examined—workshops with over 100 leaders, daily safety huddles across every inpatient unit, workspaces around quality and safety boards, end-of-year presentations where all 41 units stood up and articulated their achievements and priorities. Meaningful data was different for every team—the culture map needed to be contextualised locally rather than standardised from above. The question was never retired. The DON returned to it persistently, as her own anchor: “How do I know the care in this hospital is safe and person-centred? I keep going back to that every week.” Now, when the question is asked, it can be answered—and accreditation assessors noted that staff “don't run away from us, they run to us because they want to tell us everything they’re doing about quality and safety.”*

This index example belongs to the whole argument, not to a single programme theory. It establishes the conditions on which all subsequent findings depend: a communicative space built from organisational silence through sustained leadership, and a participatory design intent—“help it happen”—against which enactment and drift are examined across the 41 units.

### How data were used (PT1)

The tentative PT1 assumed that formal safety indicators would trigger collaborative discussion of patient safety. The empirical material revealed that the evaluation structures could be present without the collaborative mechanism firing—and that the mechanism could fire without the formal structures. The active ingredient was not the evaluation infrastructure but whether data connected to a learning-oriented culture and centred experiential alongside quantitative evidence.

Advance care planning

*A clinical leader exploring advance care planning completion rates discovered that only 1.3% of patients discharged from the hospital had an advance care plan in place—“shockingly low.” Rather than jumping to a solution, the lead spent two months in sustained inquiry: “why, why, why?” The investigation traced the problem from the hospital into residential aged care facilities, where advance care plans were not being initiated before patients were transferred to acute care. At the root was a values problem, not a systems problem—without these conversations, elderly patients were being brought to hospital “against their wishes and preferences” and dying without the dignified deaths their families wanted for them. The lead built an interdisciplinary collaboration that extended beyond the hospital walls—nursing-home managers, facility staff, and the hospital MDT working together through cycles of inquiry and action focused initially on a single facility. Completion rates rose from 1.3% to 21% within six months. The lead reflected that “80% is analysing the root” and resisted imposing a predetermined solution—“my solution could be wrong, disaster.”*

The practice gap was the starting point, but it was what the lead and team did with the data—surfacing team assumptions and values, resisting predetermined solutions, building collaboration beyond organisational boundaries—that activated PD.

A smaller-scale example illustrates the same mechanism. On one ward, staff noticed that patient falls were clustered around 7:00 a.m. The team connected this to regular furosemide administration at 6:00 a.m. They changed the timing so that more staff were available to assist with toileting, and they dramatically reduced early-morning falls. They shared the discovery with another ward, which triggered a hospital-wide change to the medication system's default settings. Hospital-wide data supported an assumption that falls occurred predominantly on night shift; ward-specific data showed the highest rates during the day. When teams were supported to interrogate their own data, they generated locally meaningful responses that no standardised falls-prevention protocol could have prescribed.

Across the programme, data transparency had a dual function: enabling learning and enacting surveillance. What counted as “data” determined which function dominated. When experiential data was centred—the culture map, patient stories, and qualitative feedback—transparency enabled collaborative learning. When quantitative performance metrics dominated—complication rates and audit compliance scores—transparency became accountability monitoring. The unit SAQ feedback, designed as a learning process, could be experienced by some staff as personal judgement on their leadership and triggered emotional responses. Monthly board photographs, or requests for “a one-pager” action plan in response to safety data, could function as compliance evidence rather than learning resources. One participant captured the tension precisely: reporting demands meant that “it shifts away from what matters to the staff on the ward.”

The shift from a punitive to a learning-oriented data culture took six years of sustained leadership. This shift is a precondition for the collaborative mechanism to operate, not an output of the evaluation structures themselves. The SAQ facilitation model—external facilitator, unit-specific data, and an appreciative framing—could create the conditions under which data served learning rather than surveillance. Where this facilitation was absent, staff became defensive and disengaged.

#### Refined PT1

IF frontline teams have access to transparent, ward-specific safety data that is facilitated for learning rather than accountability, AND the data culture has shifted from punitive to learning-oriented, AND experiential data is centred alongside quantitative performance metrics, THEN data become a shared resource for collaborative team inquiry that can challenge practice assumptions.

### How engagement was sustained (PT2)

The tentative PT2 assumed that facilitated reflection would open a communicative space. The empirical material revealed something more fundamental: communicative space must already exist—established through trust, relational safety, and mutual vulnerability—before facilitated reflection can produce its theorised outcomes. Where communicative space was absent, facilitation could produce surface-level agreement, silence, or performative engagement. Two case examples—operating through different mechanisms and producing different forms of communicative action—illuminate this finding.

Not the numbers

*In one large multidisciplinary procedural department (200 full-time-equivalent staff), the QSCF prompted the nursing leadership team to facilitate values clarification sessions across the full team—nursing, medical, allied health, and technical staff. Staff explored why they chose their profession and what mattered most to them at work. Participants who had worked alongside each other for years but never discussed these questions described feeling “seen” and “heard,” with one facilitator reporting that the emotional impact remained vivid two years later. Critically, when pressure to turn rooms over quickly and maximise procedural volume threatened quality and safety, staff drew directly on the vision statement, finding collective confidence to practise in accordance with their shared values—“we come to work to provide the best quality and safe care, not the numbers.”*

Safety huddles

*In the perioperative setting, the QSCF's safety huddle was adapted into a two-tier structure shaped by the clinical workflow. A whole-team huddle addressed operational issues—staffing, flagged patients, educational opportunities, and staff wellbeing—before a second, smaller huddle took place with the clinical team assigned to each theatre. Lists are discussed case by case with the full interprofessional team present, and concerns are raised, interpreted, and acted on in real time. For example, a RN identified a patient with incomplete preoperative assessment plans; medical and nursing staff agreed on actions together. The team leader described the design as deliberately layered—the broader huddle gave everyone “something out of it,” while the clinical huddle enabled specific interprofessional decision-making grounded in a values orientation she articulated simply: “if that were my mum or my dad, how would I like this to look.”*

These two cases show different mechanisms and outcomes. The values clarification produced a communicative space through relational means—shared vulnerability, personal disclosure, and collective meaning-making—and proved durable, being drawn on 2 years later to resist operational pressure. The perioperative huddle produced communicative space through structural means—a designed public forum with clinical common ground, interprofessional presence, and sanctioned speaking roles—but was bounded by the huddle itself. The variation within this programme theory is itself the analytical finding: communicative space takes different forms depending on how it is produced, and these forms differ in what they can sustain.

Across the broader programme, communicative space had to be built over time. Facilitators described creating valued spaces by genuinely listening—sessions felt meaningful to staff because they felt heard, even without agreement on solutions. In operating theatres, clinical common ground created immediate interprofessional dialogue that the framework's engagement strategies could not produce in settings where that common ground was absent.

Where a communicative space was not established, the engagement structures produced compliance without depth. Several observed safety huddles operated as one-directional information transfer. One participant challenged hospital safety forums directly: “Is it truly a safe space, or are we (just) saying it? … sometimes only the juggernauts, the department heads, speak out because sometimes you don't want to rock the boat.” Power hierarchies are not flattened by facilitated forums.

The nursing–medicine boundary represented a structural limit to the programme's engagement reach. Medical staff were supportive but remained largely disengaged from the framework—in part due to visiting medical officer contracting arrangements that positioned them outside the nursing-led quality and safety structures. One workshop participant observed: “whose framework is that? Oh, it's driven by nursing.” Where it did occur—in theatres, during walk-arounds—it arose from clinical common ground and shared patient risk, not framework structures. The programme achieved engagement within nursing, but the interprofessional communicative space to which PD theory aspires was achieved only where clinical necessity demanded it.

#### Refined PT2

IF communicative space already exists—through established trust, relational safety, and mutual vulnerability between leadership and teams—AND teams are facilitated to reflect on what matters locally for quality and safety, THEN staff can make their practice values visible, feel heard and valued, and shared learning becomes possible. PT2 and PT4 are explicitly coupled: PT4's contextual conditions create the communicative space that PT2 requires.

### What facilitators actually did (PT3)

The tentative PT3 assumed that clinical leads’ facilitation skills would catalyse local practice change through cycles of learning, action, and reflection. The empirical material revealed a more complex picture: the programme's facilitation pipeline—the educators, mentors, and clinical leads who translate the programme's intent into ward-level practice—primarily transmitted QI methods rather than PD facilitation. This implementation drift from the DON's intended “help it happen” towards “make it happen” was not a design failure but a fidelity problem, driven by the structural conditions under which facilitators work. Two case examples show what PD facilitation looks like when the programme's participatory intent is realised.

Critical conversations

*In the dialysis unit, a clinical lead chose to focus on advance care directive conversations—a practice challenge the team recognised as urgent but felt ill-equipped to address. Staff described feeling challenged by the emotional complexity of these discussions and uncertain about how to begin them. No protocol could resolve this—no audit, checklist, or standardised pathway could tell a nurse how to sit with a patient and ask what mattered to them at the end of life. The project required collaborative inquiry into a shared concern, building the team's collective capability and confidence over months. The NUM and CNC enabled all staff to come off the floor to attend workshops, and the clinical lead facilitated an inclusive, participatory process—taking time to build mutual understanding and unforced consensus about what actions to take rather than imposing a predetermined plan. Staff reported that the process built relationships within the team and connected them deeply to what mattered to their patients—families were being harmed by not having these discussions early, and the team was now equipped to change that. Notably, the unit's quality and safety board was visibly out of date during the observation period, yet the project carried forward precisely because it was grounded in a truly collaborative, inclusive and participatory process on the ward.*

Community glove initiative

*In the community health setting, a clinical lead identified unnecessary glove usage as a practice concern from audit data and infection control consultation. Rather than prescribing a standardised response, the lead formed a working group across dispersed community teams and deliberately chose not to delegate—“I want the staff to feel well supported when they raise issues or concerns, so they got listened to.” Staff generated their own solutions: educational resources they designed, in-service sessions recorded for colleagues unable to attend, and visual cues in every workspace linking the project to shared values. Unnecessary glove costs reduced by 40%–50%, and the project was embedded into the service's business plan. Workshop participants recognised what made it distinctive: “I decided not to tell them what to do, I actually asked them what they think.” Staff also connected the work to environmental sustainability—a source of shared meaning that gave the project legitimacy beyond clinical compliance.*

In both cases, the mechanism producing sustained engagement was the facilitative approach, not the programme's evaluation infrastructure.

Where these cases realised the DON's “help it happen” intent, the broader empirical material revealed systematic drift in the opposite direction. The programme's PD intent passes through an educator workforce trained primarily in QI methods. When educators facilitate clinical leads, they transmit what they know: audit, Plan-Do-Study-Act (PDSA) cycles, pre-post surveys, education-as-intervention. Observed clinical leads workshops focused on templates, single-page structured improvement reports, and “showing an effect.” A patient safety representative noted clinical leads’ “strong desire to jump to solutions rather than to sit and brainstorm.” In one observed SAQ meeting, facilitation was shaped more by operational demands than by collaborative reflection. The observer noted that “facilitation and its ensuing critical approaches are in danger of being co-opted to more friendly, less challenging and agreeable processes.” Facilitators were observed filling silences rather than holding space for complexity. Staff reported that “the non-negotiables (the quality processes) are very time consuming and we can't translate them into the why.”

This drift is not driven by individual choice. It is driven by organisational logic: QI produces measurable outputs that governance systems demand; PD produces relational and cultural outcomes that are harder to quantify and easier to defer. The educators are understandably transmitting what the system rewards. The governance environment that PD facilitation seeks to humanise simultaneously undermines facilitators’ capacity to do so. The distinction between facilitation and education—central to PD theory and to the DON's design intent—is collapsed in practice through the ordinary operation of the facilitation pipeline.

Where fidelity to “help it happen” was maintained, common features included locally meaningful clinical problems for which no technical QI fix was available, requiring genuinely collaborative inquiry. Facilitators intuitively operated as enablers rather than instructors. Notably, some advanced practice nurses were surprised to discover that facilitation skills mattered more than their subject knowledge when mentoring—confirming that the active ingredient is the facilitative relationship, not the clinical content.

#### Refined PT3

IF the organisation's facilitation pipeline develops and maintains genuinely collaborative PD facilitation—critical reflection, values clarification, holding communicative space—rather than defaulting to QI methods, AND facilitators are supported with protected time and skilled mentoring, THEN teams can identify locally meaningful practice priorities and work through collaborative cycles of inquiry, action, and reflection. QI tools thereby serve PD-identified purposes, not the reverse.

### What leadership created (PT4)

The tentative PT4 assumed that person-centred leadership produces continuity of purpose and democratic ways of working. However, “person-centred leadership” was underspecified. What actually sustained the programme was mutual trust and vulnerability between leaders and teams: honestly sharing constraints, being transparent about what is and isn't possible, and creating safe conditions for speaking up.

The framework provides organisational enablement for microsystem practitioner-led transformation—it names, backs, resources, and connects local improvement to purpose. This is a contextual mechanism: the meso level enables the micro level, but practitioners lead the change. Without this enablement, good practice stays local and fragile. Enablement creates space, but does not determine what fills it. Whether that space is filled by PD's participatory processes or defaults to QI compliance depends on continued investment in PD facilitation capability. Top-down structures that create space for bottom-up meaning-making generate legitimacy; top-down structures that prescribe the meaning generate compliance. Two case examples show this operating at different system levels.

Sharing is caring

*A co-director responsible for a diverse clinical programme noticed a pattern in the incident data that the individual units could not see: two wards with very different specialities were experiencing the same practice challenges—rising falls, pressure injuries, and gaps in comprehensive care—but neither recognised the shared problem. They brought the leadership teams together, presented the shared data, and proposed they work as one group. The initial response from one ward was defensive—“we don't have a problem”—while the other, having already been through a confronting data review process, told them directly: “you're starting where we started, and it hurts.” The co-director met with the combined group fortnightly, with educators, clinical nurse consultants, nursing unit managers, and patient safety officers all present. Throughout, they drew on QI tools—measurement and structured improvement cycles with timeframes—but skilled PD facilitation opened up a process of collaborative inquiry rather than mandated compliance. Rather than prescribing solutions, they enabled cross-unit observations of practice and site visits, then supported teams to generate their own action plans. Staff made changes to the care environment, developed shared risk communication tools, and initiated reciprocal education—each ward identifying what they wanted to learn from the other's clinical expertise. Over time, staff reported they had “never asked for help outside our own unit” and began speaking “as one team” rather than as separate services. The work deepened from falls prevention into comprehensive care—medication review, aggression management, environmental design—and generated person-centred innovations that staff described with visible pride. The co-director reflected that the approach was “not prescriptive—it was evolutionary over time, and the ideas came from them.”*

Ways of working

*A NUM stepping into a specialist outpatient clinic found a team that had been through repeated leadership changes and felt adrift. The initial SAQ feedback confirmed it—every domain was red or yellow. Rather than imposing immediate solutions, the NUM used the QSCF to rebuild from the foundations: co-facilitated sessions on ways of working, role clarification, and shared values. The cultural work took months. When a colleague offered to introduce patient-reported experience measures, the NUM asked her to call back in six months—“I've got to do a lot of work” first. When the team was ready, the measures transformed practice. Staff who had previously insisted they were too busy for person-centred care discovered it was more efficient to address what actually mattered to each patient. The team also redesigned the patient journey, creating an urgent access pathway so that GPs could refer newly diagnosed patients directly rather than sending them through the emergency department—reframing a potentially traumatic first encounter with a lifelong diagnosis into a supported experience. Within a year, the SAQ showed nothing in red and almost everything in green.*

The first case illustrates PT4 at the meso level. The co-director saw a pattern individual units could not see, brought them together, and sat with the uncomfortable conversations that followed—“we don't have a problem”—rather than imposing a solution. The co-director then stepped back: “the ideas came from them.” This is organisational enablement that does not prescribe what fills the space it creates. Reaching for mutual understanding and unforced consensus on how to move forward are not incidental—they are the relational conditions PT4 specifies. The second case shows PT4 at the micro level. The NUM's “call back in 6 months” is PT4's core claim enacted: cultural foundations must be built before participatory processes can operate.

Across the programme, the conditions created by leadership were consistently person-dependent rather than system-embedded. The nursing leadership whose personal relationships enabled cross-programme sharing acknowledged that “if we didn't all get on how we do, I don't think the sharing would work.” Culture maps had to be restarted when staff turnover made original visions irrelevant. One NUM captured the fragility starkly: “none of this is done by the RNs … it's mostly me.” Here the framework was sustained by individual leaders’ commitment, not distributed team ownership, leaving it vulnerable at every leadership transition.

#### Refined PT4

IF leadership at all levels enacts mutual trust and vulnerability with teams, AND the organisation provides sustained enablement for microsystem, practitioner-led transformation—naming, backing, resourcing, and connecting local practice change to shared purpose—THEN teams can maintain shared purpose through contextual disruption and the conditions for PD's core mechanisms are sustained. These conditions are currently person-dependent rather than system-embedded, making the programme vulnerable at points of leadership transition.

### How teams prioritised (PT5)

Project proliferation and improvement fatigue systematically undermined the collaborative engagement the framework depends on. One experienced participant captured the mechanism precisely: “I don't think this framework is necessarily broken, but I think it's a fantastic tool when *a local initiative* needs to be enacted to improve the service.” But when “we have that influx of regular auditing across the board … *push, push, push*, no-one engages because they don't understand how it all works together.” The assumption that more QI projects produce more safety breaks down at the point of care. The issue is not staff capacity for improvement but whether it connects to what matters.

Rise and Shine breakfast club

*On an acute aged care unit caring for long stay patients with dementia, staff initiated a “Rise and Shine Breakfast Club” that reframed mealtimes as therapeutic. Three times a week, staff blocked out 30–45 min by minimising non-essential tasks and created an inviting dining space—table setting with real cutlery, soft music. Staff sat down and ate with patients and carers. The initiative was designed using QI tools—ideas to test, integration into the morning safety huddle—but its purpose was irreducibly person-centred: the social process of sharing a meal to enhance dignity, improve nutritional intake, and trigger patients’ long-term memory. There was no expectation of participation, but over time it grew. Patients who had previously refused to leave their rooms were among the first at the table. Some started eating independently for the first time in years. Falls reduced. Staff described the change as meaningful and sustainable—they could immediately see how it connected to patient and staff experience and to their values. It did not feel burdensome. When the programme was shared with a neighbouring ward, the leader there was upset because her older facility had no physical space. The co-director's response was simple: take over the conference room. The initiative spread.*

Evidence of improvement fatigue was prevalent. Multiple participants described being overwhelmed: “having so many projects at the same time … 4 different projects on a ward at one time, so that's 4 different surveys.” Clinical leads reported doing projects “out of hours” because protected time was insufficient. Template duplication compounded the burden—one participant described receiving “another piece of paper, that is pretty much a replica of the quality board, that we have to write on a monthly thing and tick the boxes.” Parallel QI silos targeted the same clinical problem without connection.

Where prioritisation was achieved locally, engagement was deeper and outcomes were stronger. The Clinical Leads programme's own evolution from allocated portfolios (being “voluntold”) to voluntary self-selection demonstrated the same principle at the programme level. Collaborative prioritisation is not merely a practical recommendation to undertake fewer projects. It is itself a PD process—democratic, participatory, values-informed. When teams decide together what matters most, PD is enacted in the act of prioritising. When priorities are mandated from above, the collaborative process is bypassed before any project begins.

#### Refined PT5 (new)

IF frontline teams collaboratively identify a small number of quality and safety priorities grounded in local clinical meaning—the “local why”—AND leadership limits concurrent project demands while protecting time for focused improvement work, THEN teams can engage deeply with practice change through PD action cycles without project proliferation undermining collaborative engagement. PT5 is the practical counterweight to governance-driven project multiplication.

### Programme theory relationships

The five refined programme theories are not independent—they form an interdependent system (Figure 3). PT4 creates the conditions, over years, that PT2 requires and PT3 depends on. The DON's 6-year commitment to trust, transparency, and organisational enablement built the communicative space that makes participatory engagement possible and sustained the facilitation capability that PT3 demands. PT2 enables PT1 and PT3: once communicative space exists as an organisational norm, facilitated evaluation can produce collaborative inquiry rather than compliance reporting, and facilitated practice change can produce democratic cycles of learning rather than template-driven audit. PT5 creates the protected space—by limiting concurrent demands and enabling collaborative prioritisation—within which PT1 and PT3 can operate without being overwhelmed by project proliferation. PT3 determines what fills the enabled space: whether the facilitation pipeline delivers PD's collaborative processes or QI by default. This sequencing takes years, not months—consistent with the programme's evolution from establishment through embedding to maturation, where governance logics begin to reassert QI dominance.

**Figure 3:**
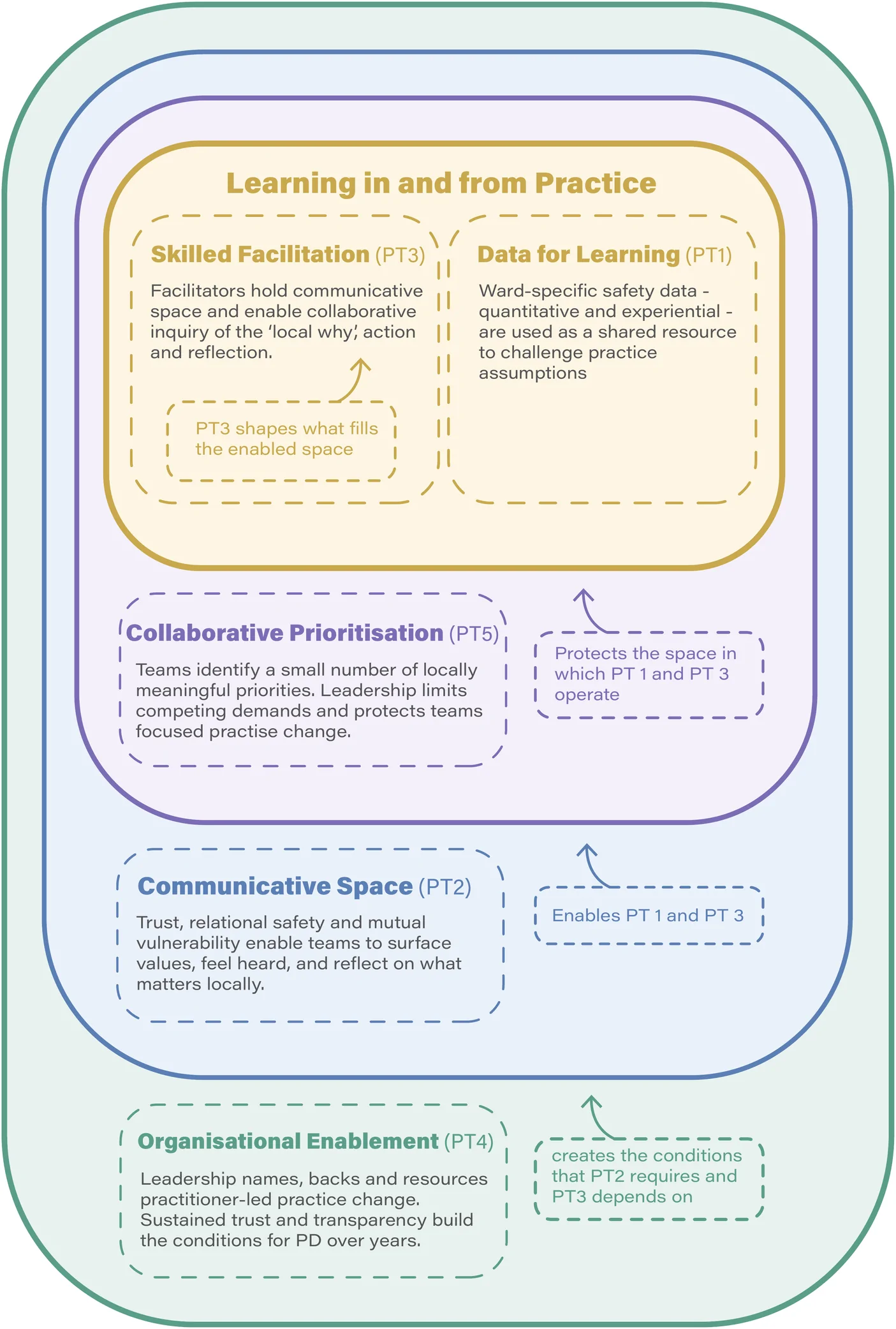
Safer Wards model: Working with Practice Development at scale. Reproduced from https://doi.org/10.6084/m9.figshare.33827089 under a CC BY 4.0 licence.

## Discussion

The QSCF successfully built what few hospital safety programmes achieve: genuine organisational enablement for practitioner-led transformation at scale. However, the evaluation reveals that organisational enablement alone does not sustain participatory practice. Five refined programme theories identify the conditions that distinguish settings where PD's mechanisms operated from settings where they did not.

In healthcare systems governed by accountability-driven standards, the participatory character of quality and safety improvement is inherently unstable (18). Governance logics continuously select for measurable outputs, standardised processes, and individual accountability. The organisational infrastructure for quality and safety improvement—educator training pathways, accreditation requirements, reporting systems, career progression—is built around QI, not PD. When organisations create space for quality and safety improvement, QI fills it by default. The implementation drift documented in PT3—from the DON's “help it happen” intent towards “make it happen” through the facilitation pipeline is driven not by choice but by organisational logic (32). This finding extends Kislov et al.'s (18) analysis of facilitation distortion by showing that the same three distortion processes operate within a programme explicitly designed to resist them. It confirms Manley et al.'s (15) warning about synecdoche: the programme's QI infrastructure was visible and measurable; its PD processes—values clarification, communicative space, collaborative inquiry—were harder to quantify, easier to defer, and more vulnerable to displacement.

Organisational enablement does not determine what fills the space it creates. Lavery (14) proposed PD and QI as allies, assuming symmetry. This study challenges that assumption. Where the facilitation pipeline delivered PD—values clarification, collaborative reflection, holding communicative space—the meso level enabled and the micro level transformed (33). Staff connected deeply to what mattered to patients and to their own values. Where the facilitation pipeline delivered technical QI by default—audit, template, education-as-intervention—the enabled space was filled with compliance structures that satisfied accreditation but did not produce the relational, cultural, and values-based outcomes that sustain safe and person-centred practice (34). Huddles could become checklists. Boards could become wallpaper. Clinical leads could become auditors.

The Venus model (3) positions PD and improvement as interdependent stems of workforce transformation. This study's contribution is to specify when that interdependence is realised rather than collapsed—when QI tools serve PD-identified purposes rather than displacing them. The five programme theories describe these conditions.

The programme theories converge with system enablers identified for large-system transformation: meaningful data, distributed leadership, authorising environments, learning cultures (12, 13). Although the QSCF had those enablers in place, yet variation persisted. What determined transformation was not the presence of enablers, but what happened in the facilitation pipeline connecting them to frontline practice (3).

### Implications

PD theory has developed robust accounts of microsystem culture change (8), effective workplace cultures (35), and the interdependent skills required for system transformation (3, 11). What it has not previously articulated is why PD's mechanisms are structurally vulnerable to displacement even when the design intent is genuinely participatory, or the ongoing conditions required for PD to operate at a hospital-wide scale in governance environments that systematically advantage QI. This study contributes five empirically derived programme theories specifying those conditions—not a one-time implementation recipe but conditions to be actively sustained against the governance logics that erode them.

These findings invite hospital leadership to shift from implementing tools and processes to shaping the relational, cultural, and organisational conditions that enable safe, person-centred practice—conditions produced not by technical interventions but by how teams engage with quality and safety in their local context:

- Be attentive to what facilitation actually transmits. When facilitation defaults to audit, PDSA, and reporting templates, participatory intent is displaced by compliance; facilitator capability for team-based critical reflection needs active maintenance as people turn over.
- Value multiple forms of evidence—and build the skill to make meaning of them. Patient and staff experience integrated with traditional safety metrics strengthens patient safety and keeps improvement anchored to what matters.
- Actively prioritise and sequence improvement demands. Project proliferation drives engagement towards compliance; helping teams locally prioritise what matters most is itself a participatory process.
- Build and sustain spaces for dialogue as a way of being, not an intervention. Grounded in team psychological safety, they serve shared understanding and coordinated action, not just risk management.
- Embed participatory principles in systems. Where the programme depended on individual relational leadership, it was vulnerable at every transition; governance, leadership development, and organisation-wide processes are what carry the conditions.

### Limitations

This is a single-site study, offering deep contextual understanding. The hospital's 20-year history of PD investment represents a distinctive contextual precondition unlikely to be present in settings implementing PD-informed programmes for the first time. Evaluation occurred during the programme's maturation phase, and findings may reflect a specific developmental stage where governance logics begin to reassert QI dominance, rather than a permanent programme characteristic. Although within-programme variation is the analytical strategy, some units may be represented more than others.

## Conclusion

The DON's foundational question—how do you know the care being provided in your unit is safe and person-centred?—inspired a 6-year programme that achieved something rare: genuine organisational enablement for practitioner-led quality and safety transformation at hospital-wide scale. However, the evaluation reveals that enablement alone does not sustain the participatory processes through which PD achieves its distinctive outcomes. The conditions specified by the five PTs must be actively maintained against governance logics that default to directive compliance.

For PD theory, this case provides an empirically grounded account of the conditions required for PD's enactment at hospital-wide scale—and of the structural vulnerability that makes those conditions necessary. For hospital leadership, the programme theories offer practical guidance: these are the conditions your programme needs, here is what they look like when enacted and when not, here is where to invest.

## Data Availability

The data generated in this study is not publicly available due to ethical restrictions. Requests to access the datasets should be directed to Clint Douglas (c2.douglas@qut.edu.au).
