# Supplementary material for: How is practitioner-led quality and safety improvement sustained across a whole hospital? A process evaluation of a practice development programme
Source: Front Health Serv. 2026 Oct 14;6:1932480. doi: 10.3389/frhs.2026.1932480 (PMC13713617; doi:10.3389/frhs.2026.1932480)
Supplement: Supplementary file 1 [file Table1.docx]

**Supplementary File 1. Interview topic guide**

- Talk about a time when you saw the programme “work” in a way that surprised you or that you hadn't anticipated? What do you think made it work in that moment?
- What have you noticed about how conversations around safety and quality might have shifted since the programme started?
- From your perspective, how do you know if the programme is making a difference? Are there ways people might overlook or not talk about?
- What are some of the unspoken rules or expectations within your team or department when it comes to safety and quality? How do these shape the way people engage with the programme?
- When you think about how the programme was facilitated, can you recall moments when things went really well, and moments when they didn’t go as planned? What did those experiences reveal about what people expect from facilitators or leaders in this context?
- Have you noticed any differences in how leadership shows up in practice, or how it’s expected to show up, based on the programme’s goals or the way it’s been implemented?
- In what ways do you think the programme has changed the way people think about their work, even in subtle ways? In what ways has it affirmed or challenged your assumptions about quality and safety?
